# Adapting developing country epidemiological assessment techniques to improve the quality of health needs assessments in developed countries

**DOI:** 10.1186/1472-6963-5-32

**Published:** 2005-04-29

**Authors:** Susan M Smith, Jean Long, Jillian Deady, Frances O'Keeffe, Deirdre Handy, Tom O'Dowd

**Affiliations:** 1 Department of Public Health and Primary Care, University of Dublin, Trinity College Centre for Health Sciences, Adelaide and Meath Hospital Dublin, Incorporating the National Children's Hospital, Tallaght, Dublin, Ireland; 2 Drug Misuse Research Division, Health Research Board, Holbrook House, Holles Street, Dublin, Ireland; 3 HSE (NA), Westward House, Russell Street, Dublin, Ireland; 4 Summer Hill Health Centre, Dublin, Ireland

## Abstract

**Background:**

We were commissioned to carry out three health assessments in urban areas of Dublin in Ireland. We required an epidemiologically robust method that could collect data rapidly and inexpensively. We were dealing with inadequate health information systems, weak planning data and a history of inadequate recipient involvement in health service planning. These problems had also been identified by researchers carrying out health assessments in developing countries. This paper reports our experience of adapting a cluster survey model originally developed by international organisations to assess community health needs and service coverage in developing countries and applying our adapted model to three urban areas in Dublin, Ireland

**Methods:**

We adapted the model to control for socio-economic heterogeneity, to take account of the inadequate population list, to ensure a representative sample and to account for a higher prevalence of degenerative and chronic diseases. We employed formal as well as informal communication methods and adjusted data collection times to maximise participation.

**Results:**

The model we adapted had the capacity to ascertain both health needs and health care delivery needs. The community participated throughout the process and members were trained and employed as data collectors. The assessments have been used by local health boards and non-governmental agencies to plan and deliver better or additional services.

**Conclusion:**

We were able to carry out high quality health needs assessments in urban areas by adapting and applying a developing country health assessment method. Issues arose relating to health needs assessment as part of the planning cycle and the role of participants in the process.

## Background

In 2001, the Department of Public Health and Primary Care in Trinity College Dublin was commissioned to carry out three health assessments in urban areas in Dublin. We aimed to carry out health assessments that would represent the needs of the whole population and not just more vocal minorities.

We were aware that we were dealing with inadequate health information systems, weak planning data and a history of inadequate recipient involvement in health service planning. International health planners have reported similar issues in developing countries and as a result, have developed appropriate health assessment survey methods. The methods for such surveys must be robust, epidemiologically sound and have the potential to collect data rapidly, inexpensively and simply [1,2]. The survey design must provide information at local level and overcome unreliable population lists. If local health care providers and communities can participate in data collection this has the potential advantage of facilitating the meeting of such needs in the local context in due course [3].

To address these issues, a thirty-cluster survey method was designed to carry out health assessments in communities in developing countries [1,4,5]. We adapted the thirty-cluster survey method and applied it in an urban setting in a developed country. The results of these three health needs assessments have been previously published [6-8] and a summary is provided in Table 1 with reference to online links to the full reports. This paper reports the adaptations made to the thirty-cluster survey method and its implementation to establish the health needs of three urban communities in Dublin, Ireland.

**Table 1:** Summary of aims and main results of the Health Needs Assessments carried out. Full reports can be downloaded from:  [see reference list for individual report links]

	Tallaght	Finglas	Docklands
	% (95% CI*)		

Aims	to assess the health needs of households and their individual members residing in each of the three areas surveyed		

Sample size	420 households 1313 individuals	420 households 963 individuals	360 households 699 individuals

Response rate	80%	77%	75%

Proportion with a chronic illness (cardiovascular and respiratory disease and arthritis most common) at the time of the survey	22% (19%–24%)	31%(28%–34%)	27% (22%–31%)
Proportion with disability at the time of the survey	3% (2%–4%)	4% (3%–6%)	3% (2%–4%)

Psychosocial issues:			
Experienced 'stress' in the 12 months prior to the survey	59% (54%–65%)	63% (57%–69%)	53% (47%–59%)
Experienced violence in the 12 months prior to the survey	10% (7%–13%)	13% (9%–17%)	11% (7%–15%)
Anxiety re: teenagers in household	60% (53%–66%)	61% (49%–73%)	64% (47%–81%)
Problem with drugs/ alcohol	2% (1%–3%)	1% (0.4%–2%)	1% (0.1%–3%)
Current smokers at the time of the survey	40% (36%–45%)	28% (24%–31%)	32% (27%–38%)

Womens' Health:			
Using family planning method at the time of the survey	56% (48%–64%)	51% (41%–60%)	46% (36%–56%)
Cervical smear in the 5 years prior to the survey	58 (52%–64%)	52% (43%–61%)	54% (46%–62%)
Breast examination in the 5 years prior to the survey	not available	47% (39%–56%)	43% (35%–51%)

Service use:			
Used hospital service in the 12 months prior to the survey	25% (22%–28%)	33% (30%–66%)	24% (20%–29%)
Attended their GP in the 12 months prior to the survey	38% (33%–43%)	57% (54%–60%)	47% (42%–51%)
Visited dentist in the 12 months prior to the survey	15% (11%–18%)	12% (10%–14%)	12% (9%–15%)
On waiting list for health care at the time of the survey	4% (3%–5%)	6% (5%–8%)	4% (2%–5%)

Identification of services needed†			
Improve out of hours GP care	52%	35%	52%
Improve services for elderly	Not identified	36%	37%
Social work services	Not identified	Not identified	25%
Services for teenagers	19%	21%	14%
Local maternity service	47%	Not identified	Not identified
Health promotion clinics	24%	31%	12%

*95% confidence intervals were calculated for the main outcome measures adjusting for strata (deprivation level), primary sampling unit (clusters) and weight (for Finglas only).

† 95% confidence intervals were not calculated for the respondents' reported health needs.

## Methods

### Design and setting

The first two health assessments were commissioned by charitable foundations seeking to support the development of health services in two separate urban areas in Dublin [6,7]. The third assessment [8] was commissioned by a local health board in another area, following the publication of the preliminary results of the first assessment. Each area contained a diverse population though there were higher deprivation levels in the area covered by the first assessment and more elderly people living in the second and third areas studied [6-8].

### Adaptations to the original model

**Figure 1:**
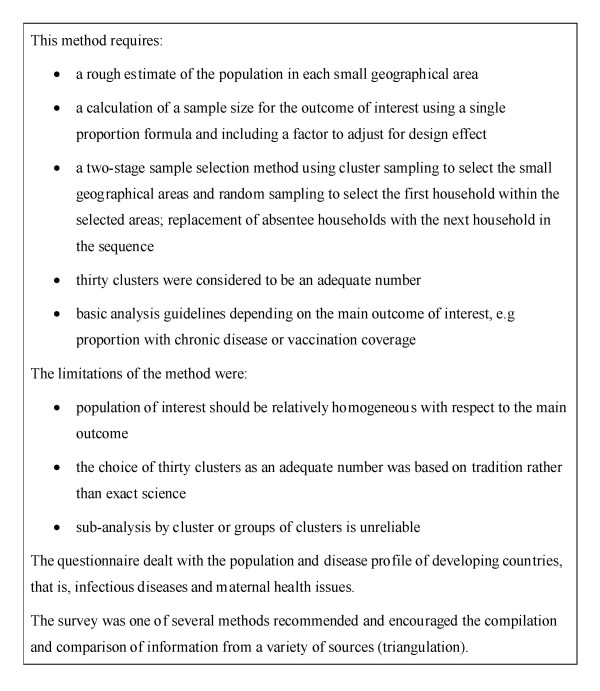
Features of model developed by the World Health Organisation, the Center for Disease Control and the Primary Health Care Management Advancement Programme to assess community health needs and service coverage [1,4,5].

**Figure 2:**
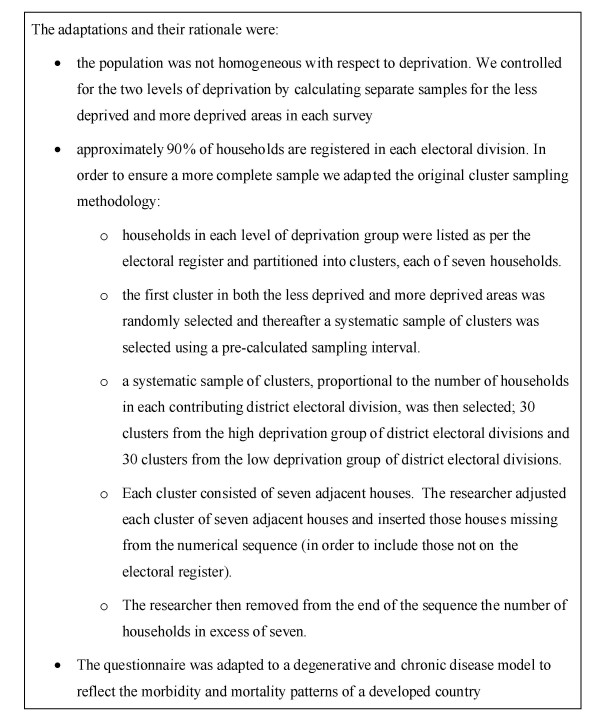
Adaptation made to model for Dublin health assessments [6,7,8].

The model was originally designed for use in homogenous populations in developing countries (Figure 1). However, the lack of socioeconomic homogeneity in urban areas in developed countries had to be considered in the selection of the study population. Detailed descriptions of the areas covered, including maps, and the slight variation in demographic characteristics in participants in each area are presented in the individual reports [6-8]. The Small Area Health Research Unit has calculated deprivation scores for all of the electoral divisions in Ireland, including the three areas surveyed (full details provided in section 2.1 of the Tallaght report [6]). These deprivation scores, based on data from the 1996 census, range from one to five depending on the level of deprivation. For each of the three areas surveyed, the deprivation scores were aggregated to form less deprived and more deprived groupings and separate samples were selected from each grouping [9]. The adaptations we made are summarized in Figure 2.

In developing countries, people are informed about surveys through community leaders and by word of mouth. We also used more formal methods, such as mailshots, posters at health and social care facilities, and advertisements in the local paper and on radio. Early on in the first survey, when many of the survey letters were returned unopened due to the high turnover of households in urban areas, we changed the addressee on survey envelopes to 'householder', giving us a response rate of at least 76% for each survey. In the second assessment, in a docklands regeneration area, access to households was difficult due to high security gates in apartment complexes, which was not considered prior to designing the survey.

## Results

### Health needs versus health service needs

The model we adapted had the capacity to incorporate health needs in the broadest sense as well as identify health service needs, and demonstrated the importance of community-based approaches to public health. The international shift to evidence based medicine has been criticised as removing the focus from the social and environmental determinants of health [10]. Some of our survey findings related to the broad determinants of health, such as the need for social outlets for teenagers and security systems for the elderly. This emphasizes the need for a multi-sectoral approach to the health of communities and stresses the importance of collecting truly representative views that reflect the social components of health. The fears expressed about personal safety led to an increased police presence on the street in one of the areas. The second assessment [7], was used by community activists to lobby the funding charity involved, to provide a bus service to transport older residents to the day centre. This focus on the broader determinants of health has also been reported in other health assessments with the development of new bus routes following such a survey in Edinburgh [11].

The reports highlight the potential discrepancy between communities' expectations of services and actual service provided as there was in fact quite high prevalence of use of hospital services. These discrepancies could be further explored using a qualitative approach. The addition of a parallel qualitative analysis of the health service providers' perspective in the second two surveys [7,8] provided more in-depth analysis of the health care services and indicated that the health service providers have a vast knowledge of the areas in which they work. Additional qualitative research involving community representatives would provide a greater depth of understanding of the inter-relationship between health needs and health care delivery needs and enable an exploration of the relationship between expectations and actual service provision.

### Community participation

The communities were consulted and encouraged to participate throughout the process. For two to three months prior to each survey, the lead researcher informed key individuals, community groups and service providers about the proposed survey and elicited their perceived needs. In each area, several health and social services needs were identified and included in the final survey questionnaires. In addition, local community members were trained and used as data collectors.

It is argued that community participation is an essential component of health needs assessment and that it should be a cyclical and iterative process in order to ensure sustainability [10]. However, sustainability is difficult as participating community data collectors are upskilled and may move on into regular paid employment. Those remaining in community work reported improved insights into local problems thus benefiting their communities.

The importance of feeding results back to the community has been previously emphasized [12]. The assessment reports were launched in public venues. We invited elected public and health board representatives, local service providers, community members and representatives of the charitable foundations involved. The community data collectors played an important role in ensuring that the wider community was aware of the launch. They actively participated in the launch by presenting their perspective on the assessments. Following the second and third assessments, summary posters were designed and placed in public areas throughout each community.

### Cost analysis

The time required to conduct each study and the cost in Euro (2001) are presented in Table 2. The cost shown for the first survey is an under-estimate as the funding did not cover the full salary costs of the lead researcher. The subsequent surveys were fully funded and allowed for a more comprehensive needs assessment including qualitative interviews with local service providers. The additional resources also allowed for wider dissemination of results, with the production of the summary posters. When compared with the cost of surveillance systems, these surveys represent good value for money. However, the addition of a qualitative component to the second and third surveys incurred an additional time and monetary cost.

**Table 2:** Time required to conduct surveys, and costs in Euro

	**First survey** **(Tallaght)**	**Second survey** **(Docklands)**	**Third survey** **(Finglas)**
Time from design to publication	April 2001 to March 2002 (11 months)	June 2001 to September 2002 (15 months)	Nov 2001 to February 2003 (15 months)

Total expenditure in Euro	32,771	96,026	91,068

### Impact of reports on services

The reports have been used by local healthcare providers, to lobby the health boards for more services, such as community antenatal clinics and a community paediatrician. The charitable foundation that commissioned the second assessment is now working with the local health board to provide a new primary healthcare centre. While it is not possible to prove conclusively that these service developments occurred solely as a result of the assessments, in each area the reports formed part of a process that directed changes in service delivery.

## Discussion

Health assessment must be practical [13] and the model we have adapted was feasible and had the advantage of encouraging community involvement. The sampling method ensured genuine representation of all community members and minimized the potential bias inherent in focusing on the priorities of more vocal minorities.

### Health needs assessment as part of the planning cycle

A favourable political environment has been described as a key component of successful health needs assessment [14]. For the two initial assessments [6,7], while the local health board approved the exercise, they had no direct role and consequently no ownership of the process. However, the first report led to another health board in Dublin commissioning the third assessment [8], which is being used to develop new services in this area. While local politicians and health board managers were briefly interested at the launch of the first report, they have not engaged further on the issues identified, which included lack of services and high levels of morbidity. By the time the third assessment was underway the environment had developed sufficiently to permit local health board involvement. As a result, the final assessment is genuinely part of the planning cycle [15] within that health board area, which has the statutory responsibility and capacity to deliver on the needs identified.

### Roles of participants in the process

Local general practitioners and other healthcare providers were involved in all three assessments although previous reports have indicated that they do not see it as part of their core activities [16].

The role of charities in identifying need is worthy, but can raise expectations which they may have no remit to address. However, the commitment of the charity involved in the second report to building a new health centre in partnership with the local health board indicates that addressing needs identified can be part of the process even for non-governmental agencies without statutory responsibility to deliver services.

The role of academic departments in the health needs assessment process might also be questioned. The charities involved perceived the department in Trinity College to be objective and unaligned with service planners and providers. Academic departments possess the expertise and capacity to carry out assessments, but might better serve the process and the communities themselves by passing on these skills. Although this would lengthen the process and increase costs initially, it would be cost efficient in the future. Issues relating to sustainability and ownership of the process arise in health services research in general and the need for linkage and exchange between planners and researchers has been highlighted [17].

## Conclusion

Our experience of using and adapting this health assessment methodology indicates the significant benefits for those seeking to carry out high quality health needs assessments in developed countries if they are prepared to learn from the experiences of developing country researchers. The adaptation of this methodology has provided more robust assessments that can be used by community members and service providers.

## Competing interests

The author(s) declare that they have no competing interests.

## Authors' contributions

SS, JL, TOD, JD and DH conceived of this paper.  SS wrote the initial draft and co-ordinated the production of the paper with the other authors.  JL conceived the methods for the needs assessment and performed the additional statistical analysis for this paper.  JL, JD, FO'K, DH and TO'D contributed to the three reports on which this paper is based.  All authors contributed to the drafting of this manuscript and read and approved the final paper.

## Pre-publication history

The pre-publication history for this paper can be accessed here:


